# Determining rhythmicity and determinism of temperature curves in septic and non-septic critically ill patients through chronobiological and recurrence quantification analysis: a pilot study

**DOI:** 10.1186/s40635-019-0267-9

**Published:** 2019-09-05

**Authors:** Vasilios E. Papaioannou, Eleni N. Sertaridou, Ioanna G. Chouvarda, George C. Kolios, Ioannis N. Pneumatikos

**Affiliations:** 1 Intensive Care Unit, Alexandroupolis University Hospital, Democritus University of Thrace, Dragana, 68100 Alexandroupolis, Greece; 2 Laboratory of Computing, Medical Informatics and Biomedical Imaging Technologies, Faculty of Medicine, Aristotle University of Thessaloniki, 54124 Thessaloniki, Greece; 3 Laboratory of Pharmacology, Faculty of Medicine, Democritus University of Thrace, Dragana, 68100 Alexandroupolis, Greece

**Keywords:** Circadian rhythm, Temperature, Complexity, Entropy, Septic shock, Critical illness

## Abstract

**Background:**

A few studies have demonstrated that critically ill patients exhibit circadian deregulation and reduced complexity of different time series, such as temperature.

**Results:**

In this prospective study, we enrolled 21 patients divided into three groups: group A (*N* = 10) included subjects who had septic shock at the time of ICU entry, group B (*N* = 6) included patients who developed septic shock during ICU stay, and group C consisted of 5 non-septic critically ill patients.

Core body temperature (CBT) was recorded for 24 h at a rate of one sample per hour (average of CBT for that hour) and during different occasions: upon ICU entry and exit in groups A and C and upon entry, septic shock development, and exit in group B. Markers of circadian rhythmicity included mean values, amplitude that is the difference between peak and mean values, and peak time. Furthermore, recurrence quantification analysis (RQA) was employed for assessing different markers of complexity of temperature signals.

Patients from group C exhibited higher temperature amplitude upon entry (0.45 ± 0.19) in relation with both groups A (0.28 ± 0.18, *p* < 0.05) and B (0.32 ± 0.13, *p* < 0.05). Circadian features did not differ within all groups. Temperature amplitude in groups B and C upon entry was negatively correlated with SAPS II (*r* = − 0.72 and − 0.84, *p* < 0.003) and APACHE II scores (*r* = − 0.70 and − 0.63, *p* < 0.003), respectively, as well as duration of ICU and hospital stay in group B (*r* = − 0.62 and − 0.64, *p* < 0.003) and entry SOFA score in group C (*r* = − 0.82, *p* < 0.003). Increased periodicity of CBT was found for all patients upon exit related to entry in the ICU. Different RQA features indicating periodic patterns of change of entry CBT were negatively correlated with severity of disease and length of ICU stay for all patients.

**Conclusions:**

Increased temperature rhythmicity during ICU entry was related with lower severity of disease and better clinical outcomes, whereas the more deterministic CBT patterns were found in less critically ill patients with shorter ICU stay.

## Background

Physiologic systems exhibit extraordinary complexity due to continuous non-linear interactions of numerous structural units and feedback loops, constituting the basic adaptation mechanism of the organism [1]. Non-linear is a term reflecting non-additive coupling of different subsystems. Recognition that physiologic time series contain hidden information related to such complexity has fueled growing interest in applying techniques from statistical physics for the study of living organisms. In this respect, we have previously shown that the analysis of continuously monitored temperature curves in critically ill patients using sophisticated techniques from signal processing theory was able to discriminate patients with systemic inflammatory response syndrome (SIRS), sepsis, and septic shock with an accuracy of 80% [2]. Similarly, Varela et al. have employed temperature signals and applied different complexity features in patients with multiple organ failure. They were able to show that reduced complexity measures were associated with increased mortality [3, 4].

Physiologic time series are not only complex but they also exhibit different rhythmic patterns in time since they fluctuate with a period of approximately 24 h. Such rhythms are called circadian and reflect synchronization of internal rhythms with external timekeepers (e.g., the change between daylight and dark, meal times). Critically ill patients experience severe circadian deregulation associated with both systemic inflammation and intensive care unit (ICU) environment [5–7]. The main circadian biomarkers are melatonin, cortisol, and temperature, whose internal rhythms are orchestrated by the “master” biologic clock located in the hypothalamic suprachiasmatic nuclei (SCN) [5].

In a recent study, we examined the potential alterations of circadian rhythmicity of urine melatonin and cortisol excretion in critically ill patients who were admitted in the ICU with septic shock or developed septic shock during their ICU stay, in a prospective fashion and on successive occasions (septic shock and recovery) [8]. We found that septic shock induced inverse changes of melatonin and cortisol circadian rhythm profiles both within and between different groups of patients, depending on the timing of onset. Furthermore, reduced rhythmicity was correlated with the severity of disease and longer ICU stay.

In this study, since circadian profiles of both melatonin and cortisol could be altered by different medications, sleep deprivation, or ICU milieu, we decided to further evaluate core body temperature (CBT) rhythmic fluctuations in the same groups of patients and with the same study design as in our previous publication. CBT and melatonin-based measurements have been considered as complementary in assessing circadian rhythms [5, 9]. Moreover, and since no study so far has evaluated a potential link between different complexity measures and circadian features of CBT signals in critically ill patients, we tried to implement recurrence quantification analysis (RQA) as a novel method of data analysis [10, 11]. RQA quantifies the number and duration of recurrences of a dynamical system presented by its state space trajectory. It has been successfully used in pilot projects in cardiology, evaluating heart rate and blood pressure regulation [12, 13]. Finally, we tried to identify potential correlations between circadian and complexity features with different clinical outcomes of interest, such as severity of disease upon entry to the ICU and both ICU and hospital length of stay. We hypothesized that altered circadian fluctuations would be significantly associated with reduced complexity of temperature curves, particularly in the more severely ill patients.

## Methods

### Study population

As we described in our previous study, subjects were recruited among critically ill patients admitted to the ICU of Alexandroupolis University Hospital, Greece, between February 2015 and July 2017 [8]. The inclusion criteria were as follows: (1) patients admitted with septic shock or those who developed septic shock during their ICU stay according to the Third International Consensus Definitions for Sepsis and Septic Shock (microbiologically confirmed infection, Specific Organ Failure Assessment score (SOFA) ≥ 2, hypotension despite adequate fluid resuscitation, plus need for vasopressor administration for maintaining blood pressure ≥ 65 mmHg, plus serum lactate > 2 mmol/L) [14], (2) age > 18 and < 80 years, and (3) ICU stay more than 48 h. Exclusion criteria were as follows: (1) history of neurological disorders; (2) history of cancer, autoimmune disease, or immune-suppressive therapy; and (3) history of recent brain injury, since such cases are commonly associated with temperature deregulation [3, 9]. Furthermore, and in accordance with Gazendam’s study, fever (CBT ≥ 38.5 °C) and/or hypothermia (CBT ≤ 36.5 °C) were also considered criteria of exclusion [9]. Mechanically ventilated patients were under stable ventilatory settings and deeply sedated with propofol and remifentanil, during CBT measurements. Vasoactive drugs and antibiotics were administered when indicated. Finally, all patients were tube-fed continuously during the day but not during the night, as has been previously described [9].

Patients were allocated into three groups: Patients who had septic shock at the time of ICU entry consisted group A (initial septic shock, *n* = 10). Group B included critically ill patients who developed septic shock during ICU stay (in-hospital septic shock, *n* = 6). Patients from groups A and B were the same as in our previous study, but we included only those who were afebrile during CBT recordings. In addition, group C (*n* = 5) included vascular surgery and trauma patients as controls, since they did not develop septic shock during their ICU stay. None of these patients received hydrocortisone or non-steroidal anti-inflammatory drugs during the whole study period. Patients during sedation had their eyes closed throughout the whole 24-h sampling period. To avoid artifacts by artificial light during night, lights were turned off during the night hours except during the nursing rounds. The ICU environment allowed regular changes between night and daylight for awaked subjects. All patients were followed until discharge from the hospital, to assess ICU and in-hospital length of stay and mortality.

### Data collection and measures

Demographic, clinical, and biochemical data were collected from the patient’s electronic medical record. Severity of disease upon admission was assessed during the first day to the ICU using both the Acute Physiology and Chronic Health Evaluation (APACHE II) score and Simplified Acute Physiology Score II (SAPS II). Furthermore, daily severity of illness was evaluated with SOFA score every morning and during the whole ICU stay, while depth of sedation was assessed once daily with the Ramsay scale.

#### CBT recordings

CBT was recorded for 24 h at a rate of one sample every 5 min, and hourly averages were computed for each subject, starting between 9.00 and 10.00 a.m. Measurements were performed with a bladder temperature sensor connected to an indwelling catheter (Rϋsch sensor series 400 Teleflex) [9]. Historically, rectal temperature has been preferred to measure a patients’ CBT. However, as has been previously shown, rectal temperature significantly lags behind other core sites, such as urinary bladder site, during acute temperature alterations [15]. Moreover, Lefrant and colleagues have found that in critically ill patients requiring a pulmonary artery catheter, urinary bladder electronic thermometer was more reliable than electronic rectal thermometer to measure core temperature with an accuracy ± 0.4 °C [15].

Entry CBT recordings were performed for all patients within less than 24 h of admission and within 24 h before discharge from the ICU. Thus, for group A, 24 h CBT measurements occurred at enrollment during septic shock (entry phase) and within 24 h before discharge from the ICU (recovery/exit phase). For group B, sampling occurred upon entry (entry phase), at the occurrence of septic shock since a diagnosis of infection was confirmed [14] (septic shock phase) and within 24 h before discharge from the ICU (recovery/exit phase). Similarly, for group C, CBT recordings were made upon entry and within 24 h before discharge from the ICU. During each 24-h study period, all patients were monitored for fever or hypothermia.

#### CBT curve analyses

The CBT curve analyses were performed as follows:

##### Circadian rhythm analysis

The circadian analysis shows whether CBT can exhibit periodicities in a 24-h scale. Briefly, this technique fits a cosine function of a fixed anticipated period to the data and approximates the following equation to experimental data, using the least squares method for minimization [16]:

1 $$ \mathrm{CBT}\ (t)=M+A\times \cos\ \left(2\mathrm{pi}\times t/24+\mathrm{acrophase}\right) $$

where *M* (mesor) is the mean level of CBT fluctuations, *A* is the amplitude which is the difference between peak and mean values, pi is 3.14159, and acrophase is the peak time that reflects the time of peak value in relation with midnight (e.g., acrophase = 0 at local midnight when the fitted period is 24 h). These three metrics are considered markers of circadian rhythmicity. The hourly CBT values were fit to this equation, and the three parameters were estimated.

The single cosinor method is appropriate for modeling the individual data when only one frequency is present; otherwise (presence of multiple periods, non-sinusoidal shape), the use of multiple components analysis is recommended [16]. The methods are related to the Fourier harmonic analysis, with Fourier analysis performed in the frequency domain and rhythmometric analysis in the time domain. Recently, the sigmoidally transformed cosine model has been proposed as a non-parametric statistic for the analysis of activity rhythms since these data resemble more closely a square wave pattern [17]. Since for some data there is no well-defined short interval of maximum values, the calculation of acrophase might be inappropriate. On the contrary, different intervals of many hours may exist during which the data are “relatively high” or “relatively low.” In this case, non-linear sigmoid transformation of the traditional cosine curves might better model biological rhythms, particularly CBT, since it can represent more accurately through rectangular waves, alternating high and low values with long time intervals (“on” and “off” states).

Nevertheless, in our case, we performed a cosinor analysis of CBT curves using the R package cosinor [18]. Briefly, the cosinor model was applied separately in each time series. In each case, it was first checked that the cosinor model estimation was valid and adequate, before adding the model parameters to the analysis. The statistics implemented in the cosinor/cosinor2 package were used for this reason [18]. First, the rhythm detection test was performed, for the significance of the estimated model for single cosinor. It calculates an *F* ratio with respect to the estimated and observed values, degrees of freedom, and the *p* value [19]. When *p* was higher than 0.05, the cosinor values were not further employed. All data passed the overall test for model significance. Subsequently, the level of correlation between model and real CBT data, as well as the percent rhythm that is the coefficient of determination obtained by squaring the correlation between the observed and estimated data, was measured [18, 19].

##### Recurrence quantification analysis

Recurrence is a term more general than periodicity, including also more irregular cyclicities, and corresponds to times at which a state of a dynamical system recurs, i.e., CBT at time *i* and *j* is very close. Starting from the visualization of recurrence, one would consider a square matrix in which columns and rows correspond to a certain pair of times, and recurrences are such (*i*, *j*) points with roughly similar CBT. Natural processes considered as deterministic dynamical systems can have a distinct recurrent behavior, with states coming close after some time. Trajectories passing from nearby paths are typical also for non-linear chaotic systems. Such recurrence of a state at time *i* at a different time *j* is marked within a two-dimensional squared matrix with one and zero dots (black and white dots in the plot), where both axes are time axes. The visual representation is called recurrence plot (RP) [10, 11]. The RPs exhibit characteristic large-scale and small-scale patterns. The first patterns are denoted as *typology* and the latter as *texture*. The typology offers a global impression and is characterized by the following:

- Abrupt changes in the dynamics or extreme events causing white areas or bands in RPs.
- Homogeneous plot for random time series.
- Oscillating systems with diagonal oriented periodic recurrent structures.

The closer inspection of the RPs reveals small-scale structures (the texture) which are *single dots* and *diagonal* lines as well as *vertical* and *horizontal* lines (the combination of vertical and horizontal lines obviously forms rectangular clusters of recurrence points):

d. Single, isolated recurrence points can occur if states are rare, if they do not persist for any time or if they fluctuate heavily.
e. A diagonal line occurs when a segment of the trajectory runs parallel to another segment, i.e., the trajectory visits the same region of the phase space at different times.
f. A vertical (horizontal) line shows a state that does not change or changes very slowly, i.e., the state is trapped for some time.

These small-scale structures are the base for recurrence quantification analysis (RQA) [20]. RQA attempts to capture and quantify the amount and distribution of points on the RP. The basis of the RQA approach is phase-space reconstruction through time-delayed embedding. A phase space is a space in which all possible states of a system under study can be charted. If full determination of the state of a system requires *N* independent variables, then the phase space has *N* dimensions. The method of time-delayed embedding allows the reconstruction of phase-space profiles from a single, one-dimensional variable, in this case CBT, according to Takens’ theorem [21]. Briefly, if a system is comprised of multiple interdependent variables and one has access only to a single observable *x* from the system (i.e., CBT), then the multidimensional dynamics of that system can be reconstructed from the single measured dimension by plotting the variable *x* against itself a certain number of times and at a certain time delay. This variable is called Takens vector’s index, and in our case, plotting was 24 number of times with a time delay of 1 h. The following features were calculated from the matrix, with NonlinearTseries R package (https://cran.r-project.org/web/packages/nonlinearTseries/index.html), as described below:

i. Recurrence (REC): percentage of recurrence points in a recurrence plot. It is a measure of the density of recurrence points in the RP and corresponds to the correlation sum.
ii. Determinism (DET): percentage of recurrence points that form diagonal lines. Processes with uncorrelated or weakly correlated, stochastic, or chaotic behavior cause none or very short diagonals, whereas deterministic processes cause longer diagonals and less single, isolated recurrence points. DET is a measure for predictability in the system. In general, deterministic systems are often characterized by repeated similar state evolution, corresponding to a local predictability.
iii. Laminarity (LAM): percentage of recurrent points that form vertical lines. LAM will decrease if the RP consists of more single recurrence points than vertical structures.
iv. Length of the longest diagonal line (Lmax): this measure is related to the exponential divergence of the phase space trajectory. The faster the trajectory segments diverge, the shorter are the diagonal lines and the higher is the measure DIV.
v. Divergence (DIV): inverse of Lmax related to the largest positive Lyapunov exponent, which is a property of complex dynamic systems and characterizes the rate of separation of close trajectories. Briefly, dynamic systems with high Lyapunov exponents exhibit fast divergence, whereas chaotic systems have the highest exponents, reflecting limited predictability of their state.

#### Statistical analysis

There were three main arms in the analysis of CBT circadian and RQA features: (1) to compare circadian and RQA parameters between different time points of measurements both between and within study groups, (2) to evaluate which metrics correlate with different clinical outcomes of interest, and (3) to reveal if there is a relation between recurrence within each 24 h period and 24 h circadian rhythmicity. A Kolmogorov-Smirnov test confirmed that CBT, age, and different scoring systems of severity of diseases followed a normal distribution. In this respect, one-way ANOVA was employed to detect differences between the three groups of patients. Regarding circadian and RQA features, as normality remained inconclusive considering the 3 groups involved, non-parametric statistical analysis via the Wilcoxon signed rank (within groups) and Kruskall-Wallis (between groups) tests was used. The package corrplot was employed for an enhanced visualization of correlation analysis based on estimation of Pearson’s *r*, between circadian and RQA features with different clinical variables, such as severity scores at entry, length of ICU and hospital stay, and mortality [22]. Positive correlations are displayed in blue, and negative correlations in red color. The color intensity and the size of ellipse are proportional to the correlation. Due to multiple comparisons and in order to protect from type I error (false-positive results), a post hoc Bonferroni correction was conducted, getting an adjusted *p* value after dividing the original *p* value (0.05) by the number of analyses performed. In our case, since we evaluated 3 circadian and 5 RQA features along with 7 clinical parameters, the new corrected *p* value was 0.003 (0.05/15). Data are presented as mean ± standard deviation. All analyses were performed with R version 3.4.4.

## Results

Five of the 15 patients from group A and 5 from the 11 patients from group B constituting the patients’ population that we studied in our previous investigation [8] were excluded from the analyses due to fever (CBT ≥ 38.5 °C). Controls from group C included three multiple trauma patients and two subjects who underwent emergency vascular surgery for abdominal aortic aneurysm rupture. From group B, one patient died in hospital due to decompensated heart failure and two patients, who suffered from multiple trauma injuries, died during ICU stay with multiple organ failure. However, CBT recordings were performed 1–2 days before death in the ICU. Since we did not perform any regression analysis in order to assess the potential impact of circadian deregulation or complexity pattern changes of CBT curves upon ICU entry on mortality, we cannot estimate the association between survival and our results. Nevertheless, entry APACHE II, SAPS II, SOFA scores, and Ramsay scale, as well as length of hospital and ICU stay, did not differ between the three groups (Table 1). In patients constituting group A, septic shock was diagnosed by the carrying physician before their transfer to the ICU and according to the Third International Consensus Definitions for Sepsis and Septic Shock (Table 1) [14]. Septic shock in patients from group B was diagnosed 7.2 ± 4.3 days after ICU admission and was attributed to ventilator-associated pneumonia (*n* = 4) and bloodstream infections (*n* = 2), according to published guidelines [23, 24]. No correlation was found between duration of illness prior to septic shock development and any circadian or RQA features.

**Table 1:** Patients’ characteristics

Parameter	Group A (*n* = 10)	Group B (*n* = 6)	Group C (*n* = 5)
Age (years)	61.2 ± 24.3	60.7 ± 14.3	62.4 ± 20.5
Diagnosis	Pneumonia (*n* = 5)	Multitrauma patients (*n* = 3)	Multitrauma patients (*n* = 3)
	Intra-abdominal sepsis (*n* = 1)	Aortic aneurysm rapture (*n* = 2)	Aortic aneurysm rapture (*n* = 2)
	Bacteremia (*n* = 3)	Acute pulmonary edema (*n* = 1)	
	Urinary tract sepsis (*n* = 1)		
APACHE II score	18.9 ± 6.2	20.3 ± 5.4	19.2 ± 4.3
SAPS II score	44.2 ± 15.6	43.5 ± 17.2	42.7 ± 14.8
Entry SOFA score	7.6 ± 3.8	7.2 ± 4.3	7.9 ± 2.5
ICU LOS	9.1 ± 8.4	12 ± 10.7	9.8 ± 10.3
Hospital LOS	26 ± 22.5	24 ± 14.1	23 ± 20.6

Differences between groups are not statistically significant. Values are mean ± SD. *APACHE II* Acute Physiology and Chronic Health Evaluation, *SAPS II* Simplified Acute Physiology Score, *SOFA* Specific Organ Failure Assessment, *LOS* length of stay

### Correlations between estimated and observed CBT data

The implementation of the single cosinor model was found to exhibit a moderate to high correlation with real CBT data. Thus, the mean and standard deviation (mean ± SD) of correlation values were as follows: (1) for group A, 0.66 ± 0.19; (2) for group B, 0.62 ± 0.19; and (3) for group C, 0.69 ± 0.19. Moreover, the percent rhythm (coefficient of determination) was as follows: for group A, 0.58 ± 0.19; for group B, 0.56 ± 0.15; and for group C, 0.59 ± 0.20, respectively.

### CBT circadian rhythm profiles

**Fig. 1:**
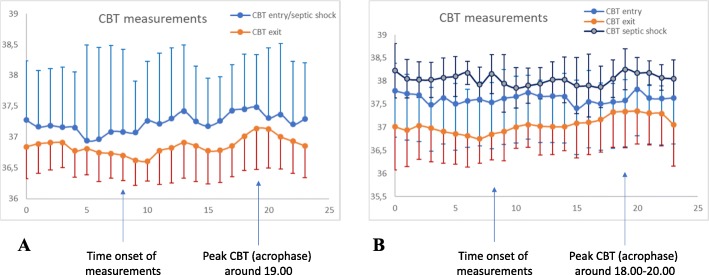
Longitudinal trends of mean core body temperature (CBT) values (*y* axis) per hour and within 24 h during different time points of measurements (*x* axis) for patients from groups A and B. **a** Linear line plots of mean CBT values along with standard deviations (SDs) in group A (initial septic shock, *n* = 10). Peak time is around 19.00 during entry/septic shock and exit whereas onset of measurements is between 9.00 and 10.00. **b** Linear line plots of mean CBT values along with SDs in group B (in-hospital septic shock, *n* = 6). Similarly, peak time is around 18.00–20.00 during entry, septic shock onset, and exit with the same time onset of recordings as in group A. The value 0 corresponds to midnight

**Fig. 2:**
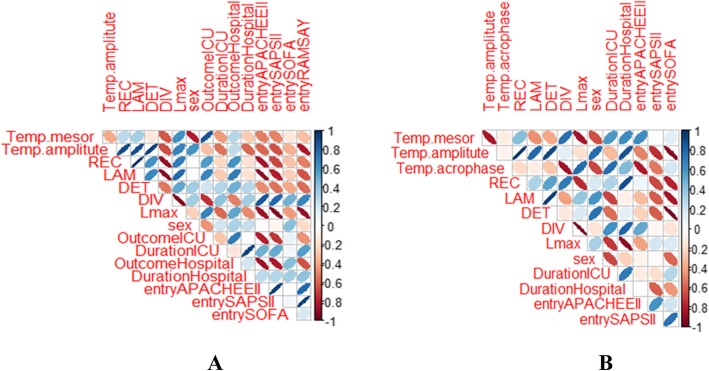
Corrplot or correlation matrix of CBT circadian and RQA metrics upon entry in the ICU, along with clinical outcomes. **a** Corrplot in group B (in-hospital septic shock, *n* = 6). Positive correlations are displayed in blue and negative correlations in red color. Color intensity and the size of ellipse are proportional to the correlation coefficients. **b** Corrplot in group C (controls, *n* = 5)

The Wilcoxon and Kruskall-Wallis tests of the hourly averages across the 24 h of CBT recordings showed no significant alterations in any circadian feature within and between groups, except for amplitude. Thus, patients from group C (controls) exhibited higher CBT amplitude upon entry (0.45 ± 0.19) in relation with both groups A (0.28 ± 0.18, *p* = 0.041) and B (0.32 ± 0.13, *p* = 0.042). Furthermore, patients from group A (initial septic shock) had reduced CBT amplitude upon entry compared to both groups B (in-hospital septic shock, *p* = 0.054) and C (controls, *p* = 0.041). All groups exhibited similar CBT peak times (acrophase) across different periods of measurements (18.00–20.00, Fig. 1a, b). CBT amplitude in groups B and C upon entry was negatively correlated with SAPS II (*r* = − 0.72 and − 0.84) and APACHE II scores (*r* = − 0.70 and − 0.63), respectively, as well as duration of ICU and hospital stay in group B (*r* = − 0.62 and − 0.64) and entry SOFA score in group C (*r* = − 0.82; Fig. 2a, b). For all correlations, *p* was less than 0.003. Such findings indicate that increased CBT circadian fluctuations during entry are related with lower severity of disease and better clinical outcomes, whereas septic shock did not seem to induce any significant deregulation on rhythm profiles of CBT within both groups A and B.

### CBT RQA profiles

**Fig. 3:**
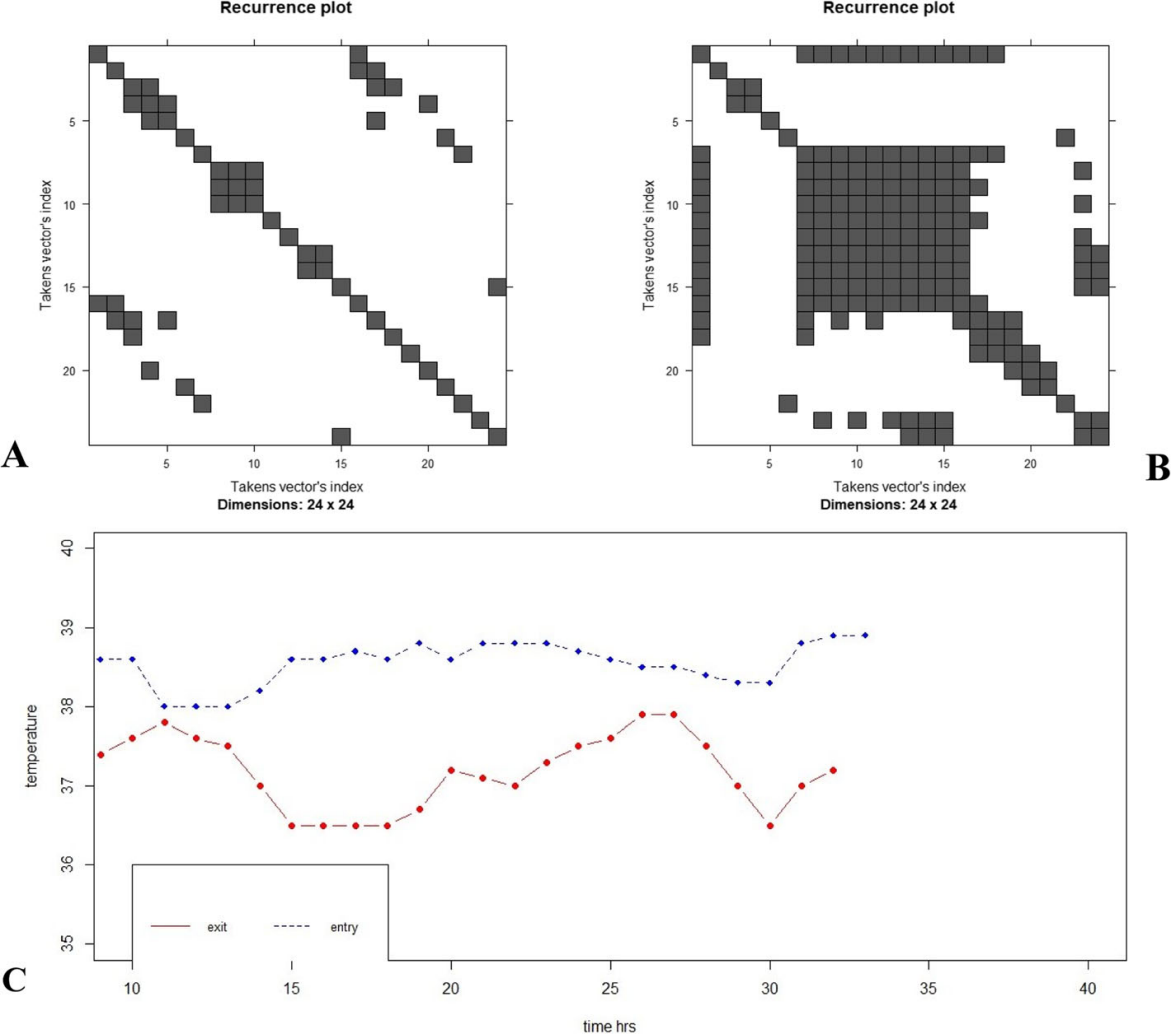
Recurrence plot (RP) of CBT curves of a patient from group A (initial septic shock). **a** Entry RP. **b** Exit RP. Both *x* and *y* axes denote time (24 h of measurements). It seems by simple inspection that upon entry, there are less diagonal and vertical lines and more single recurrence dots, whereas during exit, the combination of numerous vertical and horizontal lines forms rectangular clusters of recurrence points, indicating more periodicity of CBT curves. **c** CBT longitudinal values over 24 h. Blue corresponds to entry and red to exit. Measurements started at around 9.00 a.m. It seems that the trace during exit is less smooth and more “erratic” than that during entry. This might signal an increased rhythmicity with many fluctuations upon exit from the ICU and is associated with a more periodic pattern of CBT change in RQA analysis, depicted in **b**. Takens vector’s index in temperature as a single variable describing the systems’ dynamics and projected in a two-dimensional phase space (see text for details)

RQA metrics did not differ between the three groups of patients except for LAM, which was found to be significantly increased during exit in relation with entry values for all groups of patients (*n* = 21, 0.92 ± 0.12 vs 0.83 ± 0.23, *p* = 0.034). Thus, exit CBT curves exhibited more periodic and predictable behavior. Regarding group A, both LAM and REC were significantly reduced in entry in relation with exit (0.67 ± 0.13 vs 0.88 ± 0.07, *p* = 0.042, and 0.18 ± 0.06 vs 0.29 ± 0.16, *p* = 0.009, respectively). Furthermore, entry SOFA score was found to be negatively correlated with LAM (*r* = − 0.67, *p* < 0.003) of CBT upon entry. Such findings indicate increased probability of state recurrence and more vertical lines in exit, whereas upon entry, there are more isolated points, reflecting decreased CBT periodicity during septic shock (Fig. 3).

Considering group B, APACHE II score upon entry was found to be negatively correlated with Lmax (*r* = − 0.94), REC (*r* = − 0.92), and LAM (*r* = − 0.90) and positively correlated with DIV (*r* = 0.84) measures of entry CBT curves (Fig. 2a). In addition, DIV was positively correlated with SAPS II and entry SOFA score (*r* = 0.82 and 0.63, respectively). Such findings indicate that the more severely ill patients exhibited more single recurrence points than vertical structures and high divergence in their phase space, something that could reflect a less periodic and more chaotic behavior. Moreover, CBT amplitude during entry was negatively correlated with DIV (*r* = − 0.73) and positively correlated with DET, Lmax, and LAM (*r* = 0.73, *r* = 0.70 and 0.78, respectively), meaning that increased CBT circadian rhythmicity is associated with a more deterministic and predictable CBT profile. Finally, both ICU and hospital outcome were found to correlate with different RQA features. Thus, ICU and hospital survival were negatively correlated with DIV (*r* = − 0.82, − 0.61) and positively correlated with REC (*r* = 0.61, 0.65), Lmax (*r* = 0.72, 0.66), and LAM (*r* = 0.73, 0.75) of entry CBT signals, respectively (Fig. 2a). For all correlations, *p* was less than 0.003.

In group C, entry SOFA score was negatively correlated with CBT amplitude, DET, and LAM values upon entry (*r* = − 0.92, 0.87, and − 0.94, respectively), reflecting reduced rhythmicity and periodicity of CBT curves in more severely ill patients (Fig. 2b). Moreover, high DIV entry values were associated with increased ICU and hospital length of stay (*r* = 0.63 and 0.84, respectively). Finally, CBT amplitude upon entry was positively correlated with both DET and LAM entry values (*r* = 0.85 and 0.82, respectively). For all correlations, *p* was less than 0.003.

## Discussion

Circadian rhythms are disrupted by critical illness, patient care interactions, and unregulated light-dark patterns [5]. Such circadian deregulation usually appears with a change in amplitude of the 24-h cycle of different circadian biomarkers, such as melatonin and cortisol urine excretion or temperature curves, characterized by an almost flattened time series with reduced fluctuations. This loss of amplitude has been described as an index of impaired capacity for adaptation of the organism during stress [8, 25].

Usually, serum or urine melatonin and cortisol have been used as circadian biomarkers in different experimental and clinical studies. In addition, circadian output can be accessed through time-series profiles of CBT, as expression of central circadian pacemaker function. Circadian changes in CBT are associated to inputs from SCN upon thermoregulatory centers that modulate the set point and thresholds for cutaneous vasodilatation, involving mainly mechanisms of heat loss under resting conditions. In humans, circadian rhythms of CBT show peaks in the late evening and minima in the morning around 04.00. Daily temperature fluctuations can be affected by feeding, physical activity, and sleep, whereas with increased age, both average body temperature and the amount of its daily variability tend to decrease [26].

A few authors have evaluated CBT circadian rhythm profiles alone or in association with melatonin or cortisol, in different and heterogeneous groups of critically ill patients. Using a retrospective design, Tweedie et al. [27] characterized CBT 24-h profiles of 15 ICU patients for 8 to 26 days. They reported that 80% of all patient days had a significant circadian rhythm with substantially varying acrophases and normal amplitudes. However, the investigators did not determine if CBT parameters were related to patient condition or the ICU environment.

Nuttall et al. [28] retrospectively explored the clinical significance of circadian rhythms in 137 patients with (*n* = 17) and without (*n* = 120) ICU psychosis, by comparing for 24 h the time of both temperature and urine output nadir. They found that both groups had altered circadian rhythms and that although all “patient days” had a significant rhythm, 83% of those days had abnormal cosinor-derived parameters.

Paul and Lemmer [29] prospectively measured tympanic temperature every 1 h and plasma cortisol and melatonin levels every 2 h for 24 h, in 13 sedated ICU patients following surgery or respiratory failure and 11 patients with brain injury. They found that the 24-h circadian profiles of all measured variables were significantly disturbed, with no physiological day-night rhythm in both groups of patients in relation with healthy controls, whereas circadian rhythm alterations were more pronounced in patients with brain injuries. Additionally, although the investigators sampled each patient for 24 h and indexed each patient’s illness severity with an APACHE II score for the study day, they did not report if the patients developed sepsis or septic shock.

Pina and colleagues [6] prospectively analyzed hourly CBT and 4-h interval urine cortisol and melatonin profiles in eight burn patients for 24 h in three sessions, occurring between ICU days 1–3, day 10, and days 20–30. They also used reference values from 14 healthy young controls. Circadian rhythms of all measured variables were abolished in all patients in relation with controls.

Finally, Gazendam and coworkers [9], in a recent investigation of circadian rhythms disruption in a general ICU population, studied CBT profiles over a 48-h period in 21 patients and found significant acrophase shift in all cases (both advance and delay). Moreover, they showed that APACHE III score was significantly predictive of circadian misplacement.

In this study and using the same patients’ population from our recent publication regarding melatonin and cortisol circadian deregulation during septic shock, we tried to evaluate for the first time potential circadian disruption of CBT in patients who either were admitted with septic shock in the ICU or developed septic shock during ICU stay. In addition, we included a control group of patients who did not become septic during ICU hospitalization. We demonstrated that increased amplitude of CBT’s circadian fluctuations upon entry in patients from group B and controls was related with lower severity of disease and better clinical outcomes, such as reduced ICU and hospital length of stay.

Our findings are in accordance with those of Gazendam and colleagues regarding correlations of circadian abnormalities upon ICU entry and severity of illness, although in our case, it was the amplitude instead of the acrophase that exhibited the most significant associations. This could be attributed to the fact that in Gazendam et al.’s study, most patients were recruited 20 days on average after ICU entry [9], whereas in our study, significant correlations concerned admission CBT amplitude values, which seem to be reduced early in the course of critical illness [25].

Regarding patients’ inclusion criteria, we chose to recruit afebrile patients since both fever, fever-reducing medications, and hypothermia may mask CBT circadian rhythmicity [9]. Sedation can also affect CBT regulation and subsequently circadian rhythm profiles [2–4]. However, during ICU entry, all patients were sedated and under mechanical ventilation, whereas no differences were found in any circadian feature between the entry and recovery phase, when all subjects were awake with spontaneous respiration. Consequently, a potential impact of sedatives on our results seems unlikely.

Different time of measurements between recording periods might also affect circadian features due to a potential impact of ICU environment. However, the length of both ICU and hospital stay did not differ between all groups of patients. Furthermore, no correlations were found between the duration of illness before septic shock development and any circadian or RQA feature in patients from group B.

Although the ICU milieu might cause masking effects on melatonin profiles, CBT rhythmicity is not significantly affected by the lack of different timekeepers or other external factors that are known to influence CBT daily fluctuations [9]. Thus, physical activity and particularly upright position are nearly abolished, ambient temperature in the ICU remains more or less the same, and sleep, as has been previously described [30], is fragmented and almost evenly distributed over the whole day and night, limiting its effects on CBT rhythm profiles. However, we cannot exclude the potential impact of sleep or activity gradual restoration before discharge on our findings. Nevertheless, the lack of significant CBT circadian alterations within all groups limits the potential for such effects. Finally, food intake might affect CBT; however, all patients were tube-fed during the day, based on the same nutrition protocols [9, 31].

Inflammatory stimuli can induce a state of stress to the central nervous system (CNS), through afferent peripheral neural signaling. For instance, different pro-inflammatory cytokines produced during the acute phase of sepsis may cross the blood-brain barrier at leaky points which reduce SCN neurons’ spiking. Since SCN regulates the hypothalamic-preoptic thermoregulatory control center, a deregulation of CBT circadian profile may take place with reduced amplitude and/or a shift in acrophase [32].

In our study, patients admitted with septic shock exhibited lower CBT amplitude upon entry in relation with controls and those who developed septic shock during ICU stay. Such findings could be probably attributed to a high level of pro-inflammatory cytokines during the onset of septic shock. In addition, CBT circadian features did not change significantly within any group, although a non-significant trend toward increased values upon exit from the ICU was noticed. According to previous studies, this lack of significant longitudinal changes could be due to intra-individual variability in thermoregulation [33, 34], time-varying levels of corticotropin-releasing factor (CRF) that has been found to affect temperature fluctuations [35], or a long time needed for circadian rhythmicity restoration [36]. In this respect, Mundigler and colleagues [36] found that melatonin circadian rhythm assessment of septic patients during recovery exhibited a tendency toward a slightly restored circadian pattern in some individuals but lack of significant rhythm in any patient. Finally, the lack of significant changes between CBT circadian rhythms within all groups could be also associated with very small sample size, leading to potentially false-negative results.

Thermoregulation can be considered as a complex system, since it involves both core and peripheral temperature control mechanisms through multiple feedback loops. Loss of complexity during illness might be attributed to altered coupling between the system’s components [1]. In this respect, sepsis and critical illness have been described as a manifestation of “uncoupling of oscillators,” which are responsible for a systems’ dynamics [1, 37–41]. Different measures for assessing the complexity of biological signals, such as entropy analysis, have been investigated in different populations of critically ill patients [38]. Among different biosignals, temperature has been the less investigated system so far. We [2, 39] and others [3, 4] have found that in critically ill patients, low-temperature entropy values were associated with increased severity of disease with accuracy similar to that of SOFA.

It has been proposed that the breakdown of long-range correlations and coupling within a physiologic system may lead to either a random/chaotic or a highly regular/periodic state, and for this reason, different and multiple metrics should be used in parallel to assess the complexity of physiologic systems [1].

Non-linear methods for biological data analysis derived from chaos theory seem to be sensitive enough to uncover early phases of the disease development. Nevertheless, such calculations require large data sets. In this study, we decided to estimate CBT complexity by applying for the first time a novel non-linear method for data analysis, recurrence quantification analysis (RQA). RQA has the advantage compared to other techniques to capture the inherent dynamics of a complex system without a need of a long data series, whereas it remains relatively immune to noise [10, 11]. It has been used for evaluating heart rate variability in patients suffering from cardiovascular diseases, diabetes, and epilepsy [13, 42–44], whereas recently, its adoption as an analytical method of both heart and respiratory rate signals has been proposed, for earlier weaning outcome prediction in the ICU [45].

In our study, we found that CBT time series were more periodic upon exit and recovery in relation with entry in the ICU, for all the studying population. The more deterministic and periodic patterns of CBT, reflected in increased values of DET, LAM, Lmean, and REC from groups B and C upon ICU admission, correlated with high CBT amplitude. The more severely ill patients with longer ICU and hospital stay exhibited a more random pattern in their entry CBT signals, with increased DIV and reduced Lmean. Furthermore, in group A, an initially more random behavior upon entry was found to characterize CBT recordings, whereas before exit, more deterministic patterns seemed to occur. Similar changes were found in both groups B and C but did not reach statistical significance based on the Bonferroni criterion. Lack of sedation upon exit might constitute a potential bias on our findings; however, someone would expect increased rather than decreased randomness and divergence in awake versus sedated individuals [38, 39].

In conclusion, it seems that preserved circadian rhythmicity upon entry is associated with a more deterministic CBT profile, whereas circadian deregulation is correlated with a more random pattern of change, something that could signal impairment in the capability of adaptation to stress.

Nevertheless, our results need to be validated in different and larger data sets for standardization of the techniques involved, as well as their adoption for early and more accurate evaluation of CBT inherent dynamics during different states of stress. Moreover, RQA should be tested in association with other complexity measures in future studies.

Other limitations of our study include the small sample size that might be responsible for false-negative results (error type II), whereas multiple comparisons between different variables probably increased error type I (false-positive results). In any case, we believe that the adoption of a Bonferroni correction in our analyses has strengthened the accuracy and reproducibility of our findings. Furthermore, we consider our data set is comparable with the majority of similar studies evaluating circadian deregulation, as well as “decomplexification” in critically ill patients [40]. Finally, the absence of fever or hypothermia during CBT recordings constitutes a major limitation in terms of temperature rhythmicity and complexity assessment during critical illness and septic shock. Thus, we suggest that future studies should measure and compare circadian and complexity metrics of CBT curves in both febrile and afebrile patients, in order to evaluate possible applicability of such methods for more accurate and earlier outcome prediction of critical illness.

## Conclusions

In this study, we demonstrated that increased amplitude of CBT circadian fluctuations upon entry in the ICU of patients who developed in-hospital septic shock (group B) and controls (group C) is related with lower severity of disease and better clinical outcomes. Furthermore, the more deterministic and periodic patterns of CBT profiles upon ICU entry were correlated with high CBT rhythmicity and shorter ICU and hospital stay. Although correlations do not mean causality, the present study suggests that increased CBT randomness is related with flattened CBT curves in the most critically ill patients, in the early phases of the disease. Nevertheless, increased intra-individual and inter-individual variabilities limit the generalization of our results to different groups of patients, whereas further investigations with larger sample sizes are required to investigate the pathophysiological and potential clinical implications of our findings.

## Data Availability

The data sets used and/or analyzed during the current study are available from the corresponding author on reasonable request.

## Abbreviations

ANOVA: Analysis of variance
APACHE II: Acute Physiology and Chronic Health Evaluation II
CBT: Core body temperature
CNS: Central nervous system
CRF: Corticotropin-releasing factor
DET: Determinism
DIV: Divergence
ICU: Intensive care unit
LAM: Laminarity
REC: Recurrence
RP: Recurrence plot
RQA: Recurrence quantitative analysis
SAPS II: Simplified Acute Physiology Score II
SCN: Suprachiasmatic nuclei
SIRS: Systemic inflammatory response syndrome
SOFA: Specific Organ Failure Assessment

## References

[1] Goldberger AL, Peng CK, Lipsitz LA (2002). What is physiologic complexity and how does it change with aging and disease?. Neurobiol Aging.

[2] Papaioannou V, Chouvarda I, Maglaveras N, Pneumatikos IA (2012). Temperature variability analysis using wavelets and multiscale entropy in patients with systemic inflammatory response syndrome, sepsis and septic shock. Crit Care.

[3] Varela M, Jimenez L, Farina R (2003). Complexity analysis of the temperature curve: new information from body temperature. Eur J Appl Physiol.

[4] Varela M, Calvo M, Chana M (2005). M, Gomez-Mestre I, Asensio R, Galdos P. Clinical implications of temperature curve complexity in critically ill patients. Crit Care Med.

[5] Papaioannou V, Mebazaa A, Plaud B, Legrand M (2014). “Chronomics” in ICU: circadian aspects of immune response and therapeutic perspectives in the critically ill. Intensive Care Med Exp.

[6] Pina G, Brun J, Tissot S, Claustrat B (2010). Long-term alteration of daily melatonin, 6-sulfatoxymelatonin, cortisol, and temperature profiles in burn patients: a pre-liminary report. Chronobiol Int.

[7] Verceles AC, Silhan L, Terrin M, Netzer G, Shanholtz C, Scharf SM (2012). Circadian rhythm disruption in severe sepsis: the effect of ambient light on urinary 6-sulfatoxymelatonin secretion. Intensive Care Med.

[8] Sertaridou E, Chouvarda I, Arvanitidis K, Filidou E, Kolios G, Pneumatikos I, Papaioannou V (2018). Melatonin and cortisol exhibit different circadian rhythm profiles during septic shock depending on timing of onset: a prospective observational study. Ann Intensive Care.

[9] Gazendam JAC, Van Dongen HPA, Grant DA, Freedman NS, Zwaveling JH, Schwab RJ (2013). Altered circadian rhythmicity in patients in the ICU. Chest.

[10] Eckmann JP, Kamphorst SO, Ruelle D (1987). Recurrence plots of dynamical systems. Europhys Lett.

[11] Marwan N, Romano MC, Thiel M, Kurths J (2007). Recurrence plots for the analysis of complex systems. Phys Rep..

[12] Jr W, Zbilut JP (1994). Dynamical assessment of physiological systems and states using recurrence plot strategies. J Appl Physiol.

[13] Marwan N, Wessel N, Meyerfeldt U, Schirdewan A, Kurths J (2002). Recurrence plot based measures of complexity and its application to heart rate variability data. Phys Rev E.

[14] Singer M, Deutschman CS, Seymour CW, Shankar-Hari M, Annane D, Bauer M, Bellomo R, Bernard GR, Chiche JD, Coopersmith CM, Hotchkiss RS, Levy MM, Marshall JC, Martin GS, Opal SM, Rubenfeld GD, van der Poll T, Vincent JL, Angus DC (2016). The third international consensus definitions for sepsis and septic shock (sepsis-3). JAMA..

[15] Lefrant JY, Muller L, Coussaye JE, Benbabaali M, Lebris C, Zeitoun N, Mari C, Saissi G, Ripart J, Eledjam JJ (2003). Temperature measurement in intensive care patients: comparison of urinary bladder, oesophageal, rectal, axillary, and inguinal methods versus pulmonary artery core method. Intensive Care Med.

[16] Fernández JR, Hermida RC, Mojón A (2009). Chronobiological analysis techniques. Application to blood pressure. Philos Trans A Math Phys Eng Sci.

[17] Marler MR, Gehrman P, Martin JL, Ancoli-Israel S (2006). The sigmoidally transformed cosine curve: a mathematical model of circadian rhythms with symmetric non-sinusoidal shapes. Stat Med.

[18] Moutac. Extended tools for cosinor analysis of rhythms. Package “cosinor 2”. 2017; https://cran.r-project.org/web/packages/cosinor/cosinor.pdf

[19] Cornelissen G (2014). Cosinor-based rhythmometry. Theor Biol Med Model.

[20] Wendi D, Marwan N (2018). Extended recurrence plot and quantification for noisy continuous dynamical systems. Chaos.

[21] Takens F Detecting strange attractors in turbulence. In: Rand D, Young LS (eds) Dynamical systems and turbulence, Warwick 1981, vol 898. Lecture Notes in Mathematics, Springer, Berlin, Heidelberg

[22] Taiyun W, Simko V (2017). R package “corrplot”: visualization of a correlation matrix (Version 0.84).

[23] O’Grady NP, Barie PS, Bartlett JG, Bleck T, Caroll K, Kalil AC, Linden P, Maki DG, Nierman D, Pasculle W, Masur H (2008). Guidelines for evaluation of new fever in critically ill adult patients: 2008 update from the American College of Critical Care Medicine and the Infectious Diseases Society of America. Crit Care Med.

[24] Torres A, Niederman MS, Chastre J, Ewig S, Fernandez-Vandellos P, Hanberger H, Kollef M, Li Bassi G, Luna CM, Martin-Loeches I, J. Paiva A, Read RC, Rigau D, Timsit JF, Welte T, Wunderink R (2018). Summary of the international clinical guidelines for the management of hospital-acquired and ventilator-acquired pneumonia. ERJ Open Res.

[25] McKenna HT, Reiss IKM, Martin DS (2017). The significance of circadian rhythms and dysrhythmias in critical illness. J Intensive Care Soc..

[26] Weinert W, Waterhouse J (2007). The circadian rhythm of core temperature: effects of physical activity and aging. Physiol Behav.

[27] Tweedie IE, Bell CF, Clegg A, Campbell IT, Minors DS, Waterhouse JM (1989). Retrospective study of temperature rhythms of intensive care patients. Crit Care Med.

[28] Nuttall GA, Kumar M, Murray MJ (1998). No difference exists in the alteration of circadian rhythm between patients with and without intensive care unit psychosis. Crit Care Med.

[29] Paul T, Lemmer B (2007). Disturbance of circadian rhythms in analgosedated intensive care unit patients with and without craniocerebral injury. Chronobiol Int.

[30] Freedman NS, Gazendam J, Levan L, Pack AI, Schwab RJ (2001). Abnormal sleep/wake cycles and the effect of environmental noise on sleep disruption in the intensive care unit. Am J Respir Crit Care Med..

[31] Schibler U, Ripperger J, Brown SA (2003). Peripheral circadian oscillators in mammals: time and food. J Biol Rhythms.

[32] Esquifino AI, Cano P, Jimenez-Ortega V, Fernandez-Mateos P, Cardinali DP (2007). Neuro-endocrine-immune correlates of circadian physiology: studies in experimental models of arthritis, ethanol feeding, aging, social isolation and calorie restriction. Endocr.

[33] Lebelle PDJ, Prevot E (2001). The temperature rhythms delay of intensive care patients after surgery. Sleep.

[34] Gazendam JAC, Freedman NS (2002). The circadian rhythm of core body temperature in the intensive care unit. J Intensive Care Med.

[35] Buwalda B, de Boer SF, Van Kalkeren AA, Koolhass JM (1997). Physiological and behavioral effects of chronic intracerebroventricular infusion of corticotropin-releasing factor in the rat. Psychoneuroendocrinology.

[36] Mundigler G, Delle-Karth G, Koreny M, Zehetgruber M, Steindl-Munda P, Marktl W, Ferti L, Siostrzonek P (2002). Impaired circadian rhythm of melatonin secretion in sedated critically ill patients with severe sepsis. Crit Care Med.

[37] Pincus SM, Goldberger AL (1994). Physiological time-series: what does regularity quantify. Am J Physiol.

[38] Seely AJE, Macklem PT (2004). Complex systems and the technology of variability analysis. Crit Care.

[39] Papaioannou V, Chouvarda I, Maglaveras N, Baltopoulos G, Pneumatikos I (2013). Temperature multiscale entropy analysis: a promising marker for early prediction of mortality in septic patients. Physiol Meas.

[40] Godin PJ, Buchman TG (1996). Uncoupling of biological oscillators: a complementary hypothesis concerning the pathogenesis of multiple organ dysfunction syndrome. Crit Care Med.

[41] Goldstein B, Fiser DH, Kelly MM, Mickelsen D, Ruttimann U, Pollack MM (1998). Decomplexification in critical illness and injury: relationship between heart rate variability, severity of illness, and outcome. Crit Care Med.

[42] Mohebbi M, Ghassemian H (2011). Prediction of paroxysmal atrial fibrillation using recurrence plot-based features of the RR interval signal. Physiol Meas..

[43] Javorka M, Trunkvalterova Z, Tonhajzerova I, Lazarova Z, Javorkova J, Javorka K (2008). Recurrences in heart rate dynamics are changed in patients with diabetes mellitus. Clin Physiol Funct Imaging..

[44] Acharya UR, Sree SV, Chattopadhyay S, Yu W, Ang PC (2011). Application of recurrence quantification analysis for the automated identification of epileptic EEG signals. Int J Neural Syst..

[45] Arcentales A, Giraldo BF, Caminal P, Benito S, Voss A (2011). Recurrence quantification analysis of heart rate variability and respiratory flow series in patients on weaning trials. Conf Proc IEEE Eng Med Biol Soc.

